# A Mutational Landscape in Acute Myeloid Leukemia: Overview and Prognostic Impacts

**DOI:** 10.3390/diagnostics15192537

**Published:** 2025-10-08

**Authors:** Jeff Chen, Fares Hassan, Carlos A. Tirado

**Affiliations:** 1 The International Circle of Genetic Studies Project, Stony Brook, NY 11794, USA; jeff.chen.2@stonybrook.edu (J.C.); fares.hassan@stonybrook.edu (F.H.); 2 Department of Biology, Stony Brook University, Stony Brook, NY 11794, USA; 3 Department of Pathology, Renaissance School of Medicine at Stony Brook University, Stony Brook, NY 11794, USA

**Keywords:** acute myeloid leukemia, wild type, prognosis, mutation

## Abstract

Acute myeloid leukemia (AML) comprises 15−20% of pediatric leukemia and 35% of adult leukemia cases, requiring insights into prognostic factors of this disease to be an important aspect of diagnosis and treatment. A mutational profile of patients with AML is a crucial predictor of their outcome. Discernment of present mutations, co-mutation combinations, and variations in the mutations in a single gene requires proper research and analysis to determine their impact on a patient’s prognosis. Common and infrequent mutations are continuously investigated and analyzed in different patient cohorts, bringing new insights that lead to changes in classifications, treatments, and diagnoses. For instance, mutations in *NPM1*, *FLT3,* and *DNMT3A*, three frequent driver mutations, have high incident rates with differing prognoses and treatments in pediatric and adult patients. AML patients with *MECOM* face particularly dire outcomes, as well as those with *ASXL1* and *TP53*, making their mutational analysis crucial for review in developing a prognosis.

## 1. Introduction

AML is characterized by hematopoietic stem cell malignancies and uncontrolled immature myeloid proliferation, and it is present in 15–20% of pediatric leukemia and 35% of adult leukemia cases [1]. The disease is a complex and harsh mutational landscape that necessitates understanding its development, prognosis, and treatment strategies. Most studies of AML and its abnormalities either precede the latest European Leukemia Network (ELN) 2022 and ELN 2024 guidelines or focus on limited patient populations [2,3]. Thus, a gap remains for an updated mutational report that integrates the ELN 2022 and ELN 2024 recommendations with recent patient studies. This study uniquely addresses the gap by combining updated genomic data, pediatric and adult comparisons, and evolving guideline-based stratifications.

Firstly, it is necessary to clearly differentiate between pediatric (typically between 0 and 18 years) and adult (>18 years) AML cases, as the disease’s biology, clinical presentation, and treatment responses vary significantly between the two. Similarly, recent advancements in the treatment of AML have led to redefined risk stratifications with varying intensities of treatment. Along with the updated ELN guidelines, it has become evident that it is also necessary to differentiate between intensively treated and non-intensively treated AML in adult cases [2,3]. In pediatric AML, the disease typically presents de novo compared to adult AML, which is usually preceded by myelodysplastic syndromes (MDSs). Therapy typically is more intensive in pediatric AML than in adult AML, as pediatric AML survival rates plateaued around 60% [4,5]. On the gene spectrum, multiple differences are visible, such as *KMT2A*-AML being much more prevalent (38% vs. 2% in adult AML), or mutations in *DNMT3A* and *TP53*, common in adult AML, being almost unseen in pediatric AML [6]. In the ELN 2022 recommendations, comprehensive frameworks for risk stratification are provided for adults undergoing intense chemotherapy for AML with updates and information on genetic markers [2]. Then, in the ELN 2024 update, a separate risk stratification was introduced for patients receiving less-intensive therapies, most likely due to old age or overall health [3].

Mutations causing aberrations of cellular signaling pathways make up two-thirds of mutations in AML and consist of mutations such as *FLT3* and *KIT* [7]. *FLT3*, a receptor tyrosine kinase (RTK) expressed in immature hematopoietic cells, can express mutations involving internal tandem duplications (ITDs) and the tyrosine kinase domain (TKD). This mutation has one of the highest incidence rates in both pediatric (15%) and adult (20–35%) patients (Table 1). Meanwhile, the most common mutation in adult AML, occurring in 30–35% of cases of AML and more than 50% of AML with normal karyotypes, is *NPM1* (Table 1) [8]. *NPM1* encodes a shuttling protein for multiple purposes, including regulating histone and ribosome activity and inhibiting centrosome duplication [9]. Patients with AML-*NPM1* mutations typically have a favorable prognosis and better overall survival in the absence of FMS-like tyrosine kinase 3 mutations or *FLT3*-ITD [2]. As of the 2022 ELN guidelines, favorable risk classification mutations of AML include *NPM1* without *FLT3-ITD* and bZIP in-frame mutated *CEBPA,* intermediate risk classification mutations include *NPM1* with *FLT3-ITD,* and adverse risk classification mutations include *ASXL1, TP53, RUNX1*, etc. [2].

Cytogenetic analysis remains a cornerstone in classifying AML, as it effectively identifies large structural variants and chromosomal abnormalities, allowing patients to be categorized based on their karyotype into different prognostic groups. Each group correlates with distinct overall survival outcomes. However, modern techniques such as next-generation sequencing (NGS), the parallel sequencing of millions of DNA fragments simultaneously, have revealed novel mutations that carry their own diagnostics and prognosis [7,10]. NGS can provide a rapid read of multiple genomes or just a few genes at an incredibly accurate rate compared to earlier sequencing technologies. Furthermore, optical genome mapping (OGM), a newer tool in monitoring and detecting genomic aberrations in AML, has been shown to be even more effective in scenarios where standard cytogenetic techniques would have missed critical clinical information and recommended alternative treatments [11]. OGM provides precise observation of structural variants (SVs) by linearizing and imaging DNA through linear nano-channels and can thoroughly analyze SVs from 500 bp to multiple megabase pairs [12].

Furthermore, the presence of different genetic translocations in AML, particularly t(8;21)(q22,q22.1)/*RUNX1::RUNX1T1*, t(9;22)(q34.1;q11.2)/*BCR::ABL1*, and *KMNT2A* (MLL) rearrangements, plays a significant role in patient risk prognosis [2]. Each of these translocations is among the most recurrent and well-characterized abnormalities in AML, along with each presenting with a differing prognostic impact. As of the 2022 ELN recommendations, a t(8;21) translocation carries a favorable prognosis compared to the adverse prognosis of t(9;22) and an intermediate prognosis of *MLLT3::KMNT2A* [2]. Of these, t(8;21) is the most common, in approximately 15% of AML cases [Table 1]. The *RUNX1::RUNX1T1* fusion protein impairs the normal *RUNX1* pathway and alters early myeloid differentiation, leading to leukemic conditions with cooperating mutations, such as *FLT3* [13].

AML as a disease has a varied prognosis, depending largely on the associated mutations of each patient, as well as a limited understanding of many of its unfavorable mutations. Though some of these mutations, such as *NPM1* and *FLT3*, are fairly common, with well-studied therapeutic options, the overall genetic landscape of the disease demands further advancements and analysis.

In this review, we aim to synthesize an updated, extensive mutational landscape of AML that thoroughly reflects the adjustments from the ELN 2022 and 2024 guidelines, as well as modern technological advancements. We hypothesize that our integration of recent patient data with a discussion on current diagnostic modalities and the future of clinical care and research in the field will yield a clinically relevant framework for AML risk stratification and treatment.

We worked to complete a targeted search across PubMed/MEDLINE and Embase to inform all sections appropriately. The examination combined AML with diagnostic modalities, molecular/risk terms, and treatment concepts, with citation chaining of key papers. The inclusion of AML guidelines/consensus, meta-analyses, pivotal trials, and major studies was managed within each respective section. We excluded single-patient case reports, non-peer-reviewed items, and abstracts without full manuscripts. As a narrative review, we did not perform dual independent screening, formal quality scoring, or PRISMA registration.

**Table 1:** Incidence rate and functional groups of genetic mutations in AML by the ELN recommendations, 2024.

Mutations	Incidence Rate in AML		Functional Group
	Pediatric	Adult	
*NPM1* [1,8]	8–10%	30–35%	Nucleophosmin
*FLT3-ITD* [14]	15%	20–35%	Signaling/Kinase Pathway
*DNMT3A* [1]	2.1%	20%	DNA Methylation
*MECOM* [15,16]	<1%	4%	Transcription Factor
*ASXL1* [17,18]	10.81%	14.4–19.1%	Chromatin modification
*TP53* [1]	2.1%	10%	Transcription Factor
*CEBPA* [1]	18%	~10%	Transcription Factor
*KMT2A* [15]	10–15%	3%	Chromatin modification
*RUNX1* [1]	2.8%	10–15%	Transcription Factor
*TET2* [19]	6%	7.6%	DNA Methylation
*GATA2* [1]	2.6%	5%	Transcription Factor
*BCOR* [20,21]	1.7%	3.8–5%	Transcriptional Corepressor

## 2. Contemporary Diagnostic Modalities in AML

An accurate detection of the genetic mutations in AML is critical in guiding proper patient diagnosis, risk stratification, and therapeutic decision-making. The complexity and heterogeneity of the disease require integrated cytogenetic and molecular approaches to capture clinically relevant alterations. Furthermore, sequencing-technology advancements in the last few decades have redefined AML prognosis and classification altogether [22]. As such, many of the genetic mutations mentioned are currently detected primarily through molecular and cytogenetic testing. NGS was developed in the early 2000s but initially remained a high-cost method. In 2011, the cost of sequencing a raw megabase dropped dramatically—from around $10,000 to less than 10 cents [23]. Today, NGS, along with various forms of cytogenetic testing, serves as a primary method for identifying genetic mutations in AML. To aid method selection and interpretation, we summarize the key performance characteristics of NGS, FISH, and OGM (Table 2).

Assay choice directly affects risk assignment and therapy. Hotspot-only NGS can miss non-hotspot *DNMT3A* or atypical/long *FLT3*-ITD (or under-call allelic ratio), leading to wrong ELN/ICC risk and missed *FLT3*-inhibitor use; incomplete *IDH1*/2 or *NPM1* coverage can undercut *IDH*-inhibitor eligibility and MRD-based decisions. FISH works to rapidly confirm class-defining rearrangements (e.g., PML::RARA, *MECOM*), which helps to prevent delays in treatment and enable the movement to a higher-risk category when indicated. OGM reveals cryptic SVs (e.g., KMT2A, inv(3)), helping to refine prognosis and aid in clinical trial decisions. Accurate TP53 multi-hit status (SNV + CN/LOH) is critical; panels lacking CN/LOH or with low depth can underestimate the adverse risk.

Examples of major, commonly used myeloid NGS panels include Qiagen Human Myeloid Neoplasms Panel, Illumina AmpliSeq Myeloid Panel, Quest Diagnostics LeukoVantage Panel, and Oxford Gene Technology SureSeq myPanel (NGS Custom AML), which all provide sequencing of exons for *TP53*, *ASXL1*, *CEBPA*, *DNMT3A*, *FLT3*, and *NPM1* [22,23]. However, the extent of coverage per exon varies among platforms. The Illumina AmpliSeq Myeloid Panel provides full exon sequencing for *ASXL1*, *CEBPA*, and *TP53* but only hotspot exon sequencing for *DNMT3A*, *FLT3*, and *NPM1* [22]. It specifically focuses on 50 hotspot mutations, though this leaves room for error in non-hotspot mutations [24]. Qiagen’s panel covers regions of genes ±5–10 bases of exon/intron boundaries to allow for a broader coverage [29]. Quest Diagnostics’ panel identifies mutations in key genes, though detailed information on specific exon coverage is not as readily available, while Oxford Gene Technology offers customizable panels that allow for a variability in coverage [25,26]. Differences in these panels may introduce bias in detection and, as such, should be considered when comparing data across platforms.

For example, *MECOM* rearrangements utilize cytogenetic methods such as FISH rather than identification with NGS. *MECOM* FISH assays typically utilize break-apart probes, in which a normal cell displays the two probe signals (5′ and 3′) as fused or overlapping, producing a single signal per allele. In cells with a rearrangement, these signals split apart. A separation of the probe signals (rather than a fusion) indicates a *MECOM* rearrangement [28].

In OGM technology, it is possible to detect multiple forms of SVs to 5% Variant Allele Fraction (VAF) for mosaic/heterogeneous cancer samples, repeats, complex rearrangements, and fusions with fusion partners [12]. In comparison to FISH, it has a whole-genome coverage at a higher resolution and the ability to detect all classes of SVs in a single assay [11]. VAF cutoff value interpretation, however, cannot be universally applied to all cases. Alternative genetic events, such as duplications or multiple hits in the same gene, can impact the specificity and accuracy of VAF percentages. When clinicians consider cutoff values for VAF, it must be under the consideration of such factors. For instance, as denoted by the ICC, certain MDS cases involving *TP53* mutations typically require two mutations in a lower percentage of evaluated alleles (>10% VAF) or a single higher frequency mutation (>50% VAF). Fundamentally, the clinical significance of a VAF value depends on disease-specific guidelines as set by organizations such as the ICC [32]. Though OGM can detect multiple SVs that are typically undetectable or difficult to detect by short-read methods, short-read sequencing remains the dominant method of genetic research today due to its reliability, accuracy, and wide application [29,30].

In this review, the following sections will explore the most prevalent and comprehensively analyzed functional groups in AML, focusing on defining characteristics, impacts on disease progression, and relevance in risk factors.

## 3. *NPM1*

**Figure 1:**
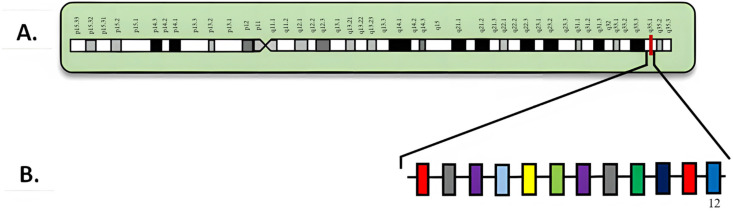
(**A**) Locus of *NPM1* on 5q35; (**B**) *NPM1* diagram with exons. Numbered exons represent typical sites of mutation. Modified from McGowan-Jordan et al., 2020 [35].

*NPM1*, located on chromosome 5q35, is the most commonly mutated gene in AML. Mutations are commonly seen in exon 12, accounting for 75–80% of these cases, and are considered an “initial step” for complete leukemia (Figure 1) [33]. Figure 1 depicts a magnified schematic of *NPM1* on chromosome 5 (Figure 1A), along with all the exons, especially exon 12, where mutations occur (Figure 1B). Wild-type *NPM1* encodes the multifunctional shuttling protein nucleophosmin that works in ribosome biogenesis, DNA repair, and cell cycle regulation. Typically, *NPM1* binds to nucleus-localized tumor suppressor protein ARF, but in mutated *NPM1*, nucleophosmin is unable to bind to ARF and is instead localized in the cytoplasm. As a result, ARF degrades and is destabilized, unable to regulate the p53 pathway. This results in an accumulation of leukemic blasts and eventually leukocytosis [34].

AML with mutated *NPM1* has been classified as a distinct entity in the fifth World Health Organization classification of hematopoietic malignancies, with diagnosis not requiring a specific percentage of blast cells [9]. Mutated *NPM1* can be identified by heterozygous mutations at the C-terminus that can cause unwinding of the three-helix bundle structure and create new nuclear export signals (NESs). Together, these changes lead to a cytoplasmic shift [33]. Patients with *NPM1*-mutated AML experience low/moderate white blood cell (WBC) counts in the absence of *FLT3*-ITD, no/low CD34, and typically favorable responses to intense chemotherapy [8].

AML with mutated *NPM1* without *FLT3*-ITD has been classified with a favorable prognosis by the ELN, though it is observed that older patients (>60 years) typically carry an inferior outcome [36]. OS is observed at 40% with complete remission (CR) around 80%. However, >50% of patients typically relapse [37,38]. A 2023 study of wild-type and mutated *NPM1* patients revealed that an *NPM1* mutation led to higher rates of CR with no effect on relapse-free survival [39]. In a 2020 pediatric *NPM1* AML study, Xu et al. saw 96.9% of patients achieving CR after two rounds of therapy and 87.7% after the first round alone. This is compared to 86.4% without *NPM1* mutations achieving CR after two rounds and 75.3% after one round. A better 5-year event-free survival (EFS) was observed in *NPM1* patients as well (66.4 ± 6.0%) compared to wild-type patients (45.5 ± 1.8%) [40]. Much more common in adult AML than pediatric AML, *NPM1* mutations in adults are very age-dependent in terms of prognostic benefit (Table 1) [41]. Patients <65 years typically experience very favorable survival outcomes, though in older patients, this benefit does not exist. In older patients, it is common to find complex karyotypes and a less intensive therapy treatment [41]. In general, non-intensively treated patients with *NPM1*-mutated AML do not see a prognostic benefit as beneficial, especially in terms of OS, compared to intensively treated patients [42]. Overall, *NPM1* in AML is characterized by a higher remission rate following intensive induction chemotherapy, although its prognosis in long-term trials is variable [43].

Instances involving *FLT3*-ITD and/or *DNMT3A* mutations often occur, and patients with these three simultaneous mutations portray a poor prognosis. Oñate et al., in 2022 [44], found that *DNMT3A* mutations appeared in 73% of AML-*NPM1* patients, along with a correlation of *DNMT3A* mutations in younger patients. Additionally, a separate 2022 study by Borlenghi et al. [45] found that 19.3% of 366 AML patients carried both *NPM1* and *FLT3* mutations. *NPM1*-mutated patients made up 40.3% of the patients with a CR rate of 93.9% and 3-year and 5-year OS rates of 72% and 64%, respectively. In this study, AML patients with *NPM1*, *FLT3*, or both were treated for over 17 years with an idarubicin-based induction regimen, followed by the addition of idarubicin to high-dose cytarabine (HD-ARAC) for consolidation. Compared to *NPM1*-mutated patients who received conventional chemotherapy, those treated with this regimen fared significantly better.

In multiple studies, it has been shown that venetoclax with or without a hypomethylating agent (HMA) is an effective treatment for *NPM1*-mutated AML, with high response rates and durable remissions [46,47]. Venetoclax by itself can target the *NPM1* apoptotic pathway through the inhibition of B cell leukemia (*BCL-2*) [46]. Though recent drugs in *FLT3* and *BCL-2* inhibitors have recently been approved, the standard treatment remains standard induction “7 + 3” chemotherapy with or without *FLT3* inhibitors and consolidation with high/medium doses of cytarabine [48]. Menin inhibitors, which target *HOX* and *MEIS1* genes and disruption of the menin-*KMT2A* complex that led to leukemogenesis, have been shown, in recent clinical trials, to have promising results in response rates [49]. Similarly, they are still in clinical development, and XPO1 inhibitors have also been seen to downregulate *HOX* genes [50]. In one study, co-treatment of both menin and *XPO1* inhibitors showed an improved increase in apoptotic cell death compared to either agent alone [51].

## 4. *FLT3*

**Figure 2:**
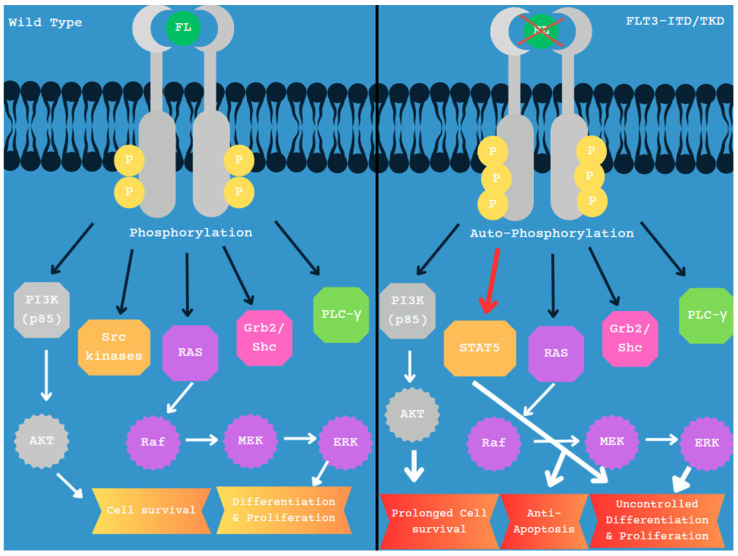
Wild-type vs. mutated *FLT3.* Black arrows indicate direct interaction with the *FLT3* protein, and white arrows indicate downstream effects and related pathways. STAT5 proteins are not as effectively activated by wild-type *FLT3* and are not substantially observed. *FLT3*-ITD mutated protein efficiently activates STAT5 and is therefore included on the right panel, with a red arrow designation. STAT5 increases transcription of Cyclin D1, c-Myc, p21, Pim-1, and Pim-2, leading to factors for anti-apoptosis and uncontrolled proliferation, as outlined in panel 2 [53].

*FLT3* (fms-like tyrosine kinase 3), located on 13q12 encodes for a transmembrane RTK belonging to the class III family of RTKs. Normal activation of *FLT3* plays an important role in several cellular processes, including phospholipid metabolism, transcriptional regulation, cell proliferation, apoptosis, and maintaining a close association with the RAS signaling pathway [52]. Wild-type *FLT3* physically associates with PI3K, the RAS GTPase, Grb2/shc, PLC-γ, and SRC kinases, leading to downstream signaling, resulting in controlled hematopoiesis, regulated proliferation, and normal cell survival (Figure 2) [53]. Mutations in the *FLT3* gene create a protein that lacks regulation and participates in autophosphorylation, leading to enhanced signaling and irregular activation of STAT5, which activates transcription for Cyclin D1, c-Myc, p21, Pim-1, and Pim-2; these irregularities cause uncontrolled proliferation in AML and prolonged cell survival (Figure 2) [53].

**Figure 3:**
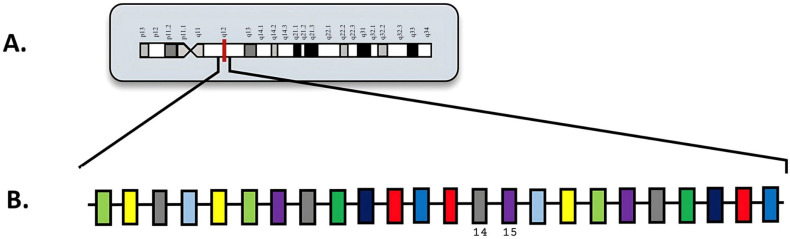
(**A**) Locus of *FLT3* on 13q12; (**B**) *FLT3* diagram with exons. Numbered exons represent typical sites of mutation. Modified from McGowan-Jordan, et al., 2020 [35].

*FLT3* internal tandem duplication (*FLT3*-ITD) mutations are characterized by in-frame insertions of duplicated sequences within exons 14 and 15 (Figure 3) [54]. In Figure 3, exons 14 and 15 are visualized in a larger schematic (Figure 3B) involving all the exons in *FLT3*, as well as an overview of chromosome 13 with key locations labeled (Figure 3A). These mutations allow for uninterrupted activation of the protein by negatively affecting the tyrosine kinase domain. As a result, *FLT3*-ITD mutations promote autophosphorylation of the tyrosine kinase domain, which induces continuous proliferative signaling in immature hematopoietic cells [54]. This dysregulation can contribute to hyperleukocytosis, which is an abnormality commonly observed in AML patients. Hyperleukocytosis is defined by elevated WBC counts, typically above 100,000 cells/µL, although counts above 50,000 cells/µL can also have significant clinical implications and likely require immediate medical intervention. Furthermore, *FLT3*-ITD mutations have been consistently associated with adverse prognostic outcomes in AML, corresponding with increased disease aggressiveness and higher relapse rates.

The second type of mutation that can occur in the *FLT3* gene is *FLT3* tyrosine kinase domain (*FLT3*-TKD) mutations. These are typically caused by point mutations at codon D835 or deletions at codon I836 within the activation loop of the *FLT3* receptor. These *FLT3*-TKD mutations disrupt the autoinhibitory mechanisms of the protein, resulting in its constitutive activation and subsequent increased proliferation of immature hematopoietic cells [55]. In reference to the prognostic implications, *FLT3*-TKD mutations are generally associated with a more favorable outcome in patients with AML compared to those with *FLT3*-ITD mutations [55]. However, the overall prognostic significance of *FLT3*-TKD mutations remains a topic of ongoing debate among researchers and clinicians.

A multivariable study by Shimony et al. (2023) [56] examined the overall survival outcomes of patients aged 60 to 75 years with AML, comparing those with *FLT3*-ITD mutations (*FLT3*-ITD+) to those without (*FLT3*-ITD-). The cohort consisted of 378 patients; however, only 53 were identified as *FLT3*-ITD+. The study found that *FLT3*-ITD+ patients did not experience significant differences in overall survival when treated with either intensive chemotherapy or HMAs [56]. Furthermore, a 2022 study by Konopleva et al. [57] ratified the efficacy of venetoclax by analyzing its effectiveness in combination with azacitidine in patients with *FLT3*-mutated (*FLT3*mt) versus wild-type (*FLT3*wt) AML. The findings indicated that the overall survival for the *FLT3*wt group receiving venetoclax and azacitidine was 12.5 months, in comparison to 8.6 months for those who received only azacitidine. In the *FLT3*mt group, the overall survival was reported as 14.7 months with venetoclax and azacitidine versus 10.1 months with azacitidine only [57]. These results provide evidence of the success of incorporating venetoclax into treatment regimens, especially when compared to azacitidine alone. They also demonstrate comparable overall survival outcomes between the *FLT3*mt and *FLT3*wt groups undergoing similar treatments.

The first *FLT3* inhibitor, midostaurin, showed significant improvement in the prognosis of *FLT3* patients, helping move *FLT3*-ITD mutations from an adverse risk classification in the 2018 ELN guidelines to an intermediate risk classification in the 2022 ELN guidelines [2,58,59]. In general, *FLT3* inhibitors are now standard in initial therapy for *FLT3* mutant AML patients and followed by consolidation with intermediate-dose cytarabine. Second-generation inhibitor, quizartinib, has been studied to have an improved median OS as well as being a generally well-tolerated and effective treatment in newly diagnosed *FLT3*-mutated AML patients [59]. Meanwhile, for non-intensive-treatment patients, combining *FLT3* inhibitors with HMAs has been standard, with improved CR rates and negative molecular residual disease (MRD) [60]. However, with the introduction of several *FLT3* inhibitors, drug resistance has also been well documented. The clonal evolution of *FLT3*-ITD mutations seems responsible for resistance and disease progression in particular. As seen in one study by Stone et al., 59% of patients treated with midostaurin achieved CR, after which half of them relapsed [60].

## 5. *DNMT3A*

**Figure 4:**
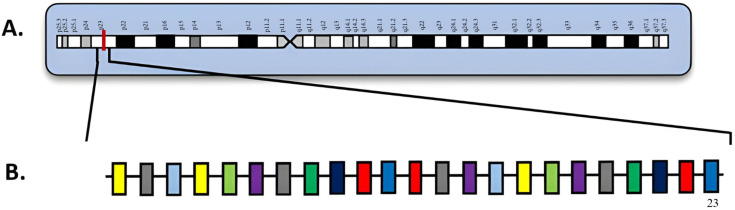
(**A**) Locus of *DNMT3A* on 2p23; (**B**) *DNMT3A* diagram with exons. Numbered exons represent typical sites of mutation. Modified from McGowan-Jordan, et al., 2020 [35].

*DNMT3A* is located on chromosome 2p23 and encodes for DNA methyltransferase 3 alpha, a DNA methylation protein that plays a key role in embryogenesis and gene regulation in de novo methylation. It is especially important for CpG methylation, which is critical during embryonic development. (Figure 4). By encoding a DNA methyltransferase enzyme to regulate gene expression, hematopoietic cell differentiation is guided primarily into granulocytes [61]. Meanwhile, *DNMT3A* mutations have been observed in 20% of AML cases and 30% of cytogenetically normal AML (CN-AML) cases, with a frequent association with a lack of tumor suppressor cell silencing typically present in standard DNA methylation [61]. The *DNMT3A*-R882 missense mutation is the most common mutation in *DNMT3A*, accounting for about 60% of cases. In Figure 4, R882 is present on exon 23 (Figure 4B) of DNMT3A with its location on chromosome 2 (Figure 4A). *DNMT3A*-R882 missense mutations often occur alongside *NPM1* and/or *FLT3* mutations. This mutation typically prevents methyltransferase activity/DNA binding and hematopoietic activity, resulting in focal methylation at CpGs in the genome [62,63,64]. In 2020, Bezerra et al. [63] observed 507 patients with de novo AML, of which 64 (13%) were identified with a *DNMT3A*-R882 mutation. Of these 64, 35 patients (~55%) were “triple-mutated,” meaning they had mutations in *NPM1*, *FLT3*, and *DNMT3A*. This group represented about 7% of the total 507 patients. Furthermore, the age group 41–60 years had the highest percentage of *DNMT3A* patients, with 34 of the 64 *DNMT3A* patients (53.1%) and 21 of the 35 (60%) “triple-mutated” patients.

The prognostic impact of *DNMT3A* mutations occurring with *NPM1* and *FLT3* mutations has had conflicting outcomes in past findings. Due to *DNMT3A* mutations typically occurring in patients presenting with *NPM1* and *FLT3* mutations simultaneously, which are very telling predictors of patient prognosis, the independent prognostic impact of this mutation is oftentimes misrepresented or underestimated [65]. A 2023 analysis found high levels of *DNMT3A* mutations in *NPM1*mut AML patients, with 47.7% of a 174-patient cohort showing these mutations. Within this group, the *DNMT3A*-R882 mutation was identified as a strong predictor of poor prognosis, as it was associated with shorter overall survival [65]. This study failed to highlight any clinical significance of *DNMT3A* non-R882 mutations.

Additionally, *DNMT3A* plays a vital role in clonal hematopoiesis (CH), an age-related disease that appears frequently in the average population [66]. In clonal hematopoiesis of indeterminate potential (CHIP), patients with a single DNMT3A mutation have the lowest risk of progression to myeloid neoplasms [67]. Given their frequent status as preleukemic clones, patients with *DNMT3A* mutations may also require additional monitoring for minimal residual disease (MRD). In one 2022 study of AML-*NPM1* patients, those with tandem *DNMT3A* mutations were all MRD-positive after the first cycle of chemotherapy and had significant *DNMT3A* persistence in further cycles [44]. However, *DNMT3A* mutations commonly remain detectable in patients even after successful treatment, leading to false MRD positives. Instead, it may be an indication of maintained CH and not relapse/refractory disease. As of the ELN 2022 guidelines, it has been recommended to exclude mutations such as *DNMT3A*, *TET2*, and *ASXL1* (DTA) from MRD assessment, given their prevalence in CHIP carriers [68,69]. *TET2* appears in 7.6% of adult AML patients, typically as loss-of-function variants, and when occurring with *ASXL1*, is generally linked with an adverse prognosis (Table 1) [19,70]. *TET2* itself has a typically adverse outlook, with a 2025 study finding a shorter median OS, lower CR rates, and a decreased response to allo-HSCT treatment [70].

## 6. *MECOM*

**Figure 5:**
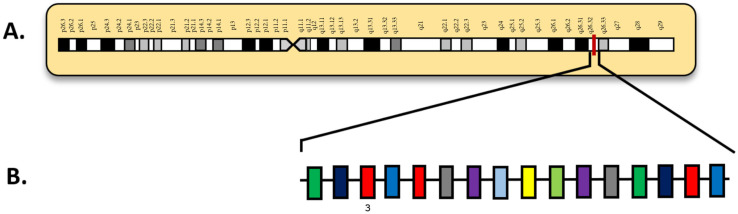
(**A**) Locus of *MECOM* on 3q26.2; (**B**) *MECOM* diagram with exons. Numbered exons represent typical sites of mutation. Modified from McGowan-Jordan, et al., 2020 [35].

Appearing in approximately 4% of AML patients, rearrangements of the myelodysplasia syndrome 1 (*MDS1*) and ecotropic viral integration site 1 (*EVI1*) complex locus (*MECOM*) are rare but notable [15,71]. Located on chromosome 3q26.2, *MECOM* encodes a protein involved in transcriptional regulation with roles in hematopoiesis, apoptosis, and cell differentiation (Figure 5). In Figure 3, exon 3 is highlighted as the location of the first full in-frame ATG start codon (Figure 5B) and its location on chromosome 3 (Figure 5A). As an oncogene, mutated *MECOM* has a very unfavorable prognosis and is heavily linked to bone marrow fibrosis and thrombocytopenia [2]. *MECOM* rearrangements (*MECOM*-R) are a critical biomarker in prognosis in AML and a defining genetic abnormality in AML regardless of blast count by the fifth World Health Organization classification of hematologic malignancies [2]. *MECOM* translocations at 3q26.2 are well represented in the literature surrounding myeloid disorders, including AML, MDS, and chronic myelogenous leukemia (CML) [72].

Mutations inv(3)(q21q26.2) and t(3;3)(q21;q26.2) occur in 30–50% of *MECOM*-R in AML. These cause the super-enhancer of GATA-binding protein 2 (*GATA2*) found at 3q21 to be affected by *MECOM* at 3q26.2, leading to transcriptional errors [15]. Otherwise, *MECOM*-R can be associated with a variety of possible chromosomal abnormalities, which are collectively known as “atypical 3q26.2/*MECOM*-R” [15].

Chantana et al., in 2023 [71], observed *MECOM* rearrangements in AML/MDS patients, comparing wild-type and mutated *MECOM* patients who were subsequently categorized into favorable, intermediate, and adverse prognosis subgroups. It was found that AML patients with *MECOM* mutations experienced low WBC counts and, interestingly, high platelet counts. Furthermore, a female predominance was observed in this group. Among the six patients studied, the 1-year survival rate was 16.7%, 0% CR, and no survivors after two years. In recent cases, *MECOM* rearrangements have also been documented in lymphoid disorders, such as acute lymphoblastic leukemia (ALL) and persistent polyclonal binucleated B-cell lymphoma (PBBL) [72]. Overall, *MECOM* rearrangements remain relatively rare, particularly in the context of lymphoid malignancies. More research and documentation are needed, especially in areas such as therapeutic approaches and treatment strategies.

## 7. *ASXL1*

**Figure 6:**
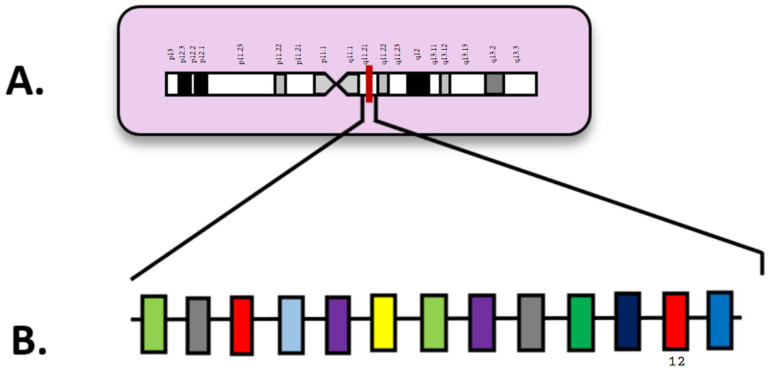
(**A**) Locus of *ASXL1* on 20q11.21; (**B**) *ASXL1* diagram with exons. Numbered exons represent typical sites of mutation. Modified from McGowan-Jordan, et al., 2020 [35].

ASXL transcription regulator 1 or *ASXL1* (additional sex combs like 1), located on chromosome 20q11.21 (Figure 6), is a gene that normally provides instructions for proteins involved in chromatin remodeling. As seen in Figure 6, exon 12 is highlighted as the most frequently mutated exon (Figure 6B) in *ASXL1* on chromosome 20 (Figure 6A), especially those with intermediate-risk karyotypes [73]. Through this mechanism, *ASXL1* plays a role in the regulation of *HOX* gene expression through activation and repression. In acute myeloid leukemia, *ASXL1* mutations typically occur as somatic nonsense or insertion/deletion mutations adjacent to the plant homeodomain (PHD) [74]. Homozygous frameshift/nonsense mutations of the gene are shown to completely silence the protein expression, as demonstrated by a Western blot analysis [74].

*ASXL1* mutations have a relatively high incidence rate of 10.8% in adults and 14.4–19.1% in pediatric patients (Table 1). Though *ASXL1* is typically associated with a worse prognosis, due to the mutation’s role in gene dysregulation triggering the occurrence of AML, this notion is still considered relatively controversial. AML with mutant *ASXL1* is shown to have a worse 5-year overall survival and event-free survival (EFS) in patients presenting with a WBC count 50 × 10^9^/L [17]. However, patients receiving hematopoietic stem cell transplantation experienced both a higher 5-year overall survival and EFS [17].

A 2023 analysis with a sample of 219 adult AML and MDS patients showed that >94% of patients present with mutant *ASXL1* had concomitant mutations, noting that wild-type *NPM1*, *FLT3*, and *CEBPA* were associated with the presence of mutant *ASXL1* [42]. The presence of *ASXL1* mutations implicated an overall worse CR rate in patients. Notably, co-mutations with *RUNX1* were linked to an even worse prognosis [75]. *RUNX1* mutations themselves are typically associated with a poor prognosis, lower CR, and OS [31]. *RUNX1::RUNX1T1*, from t(8;21)(q22;q22.1), occurs in 10–15% of adult AML but is associated with a favorable prognosis as well as being a key MRD marker [1,2].

A 2022 meta-analysis by Zhang et al. (2023) [76] reviewed studies from three databases (PubMed, Embase, and Cochrane Library), in which a final 10 studies were included in the analysis regarding *ASXL1* mutations in AML. Across all analyzed studies, patients with AML and *ASXL1* mutations consistently showed lower overall survival. The prognostic impact of *ASXL1* mutations was especially significant in patients with cytogenetically normal AML (CN-AML), with a hazard ratio of 1.78 for overall survival at a 95% confidence interval [76]. It was also indicated that patients with MDS and *ASXL1* mutations were more likely to develop AML and that these mutations occurred more frequently in patients with an older age of onset [76].

## 8. *TP53*

**Figure 7:**
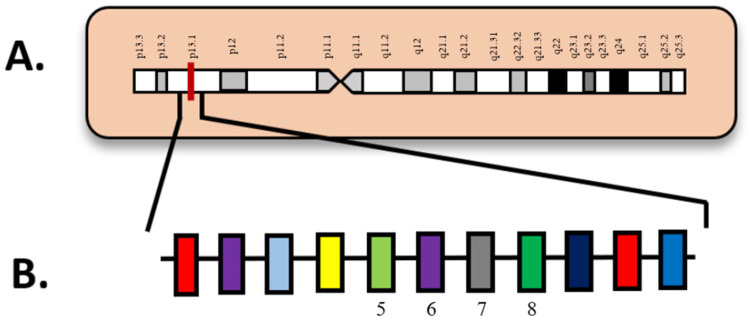
(**A**) Locus of *TP53* on 17p13.1; (**B**) *TP53* diagram with exons. Numbered exons represent typical sites of mutation. Modified from McGowan-Jordan, et al., 2020 [26].

**Figure 8:**
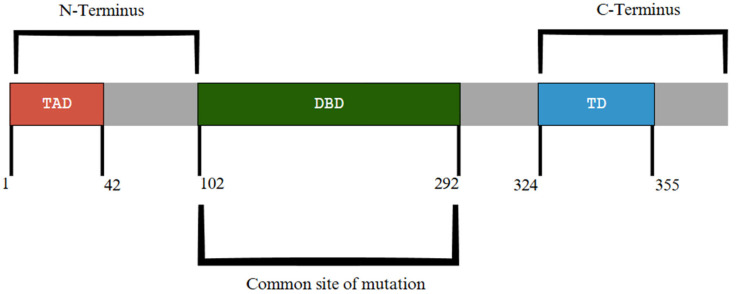
Relative domain locations in p53 protein, with amino acid range for each domain presented. Location of DNA-binding domain (DBD), in which most mutations occur, and carboxy-terminal tetramerization domain (TD), with amino-terminal transactivation domain (TAD) located at opposite N-terminal end [77]. Modified from Chen et al., 2022 [77].

*TP53* (tumor protein 53), located on chromosome 17p13.1, encodes the tumor protein p53, which serves a critical role in regulating genomic stability and tumor suppression, as seen in Figure 7A Further represented in Figure 8, exons 5–8 of *TP53* encode the DNA-binding domain where the majority of *TP53* mutations occur (Figure 8) [77]. Other functional domains of p53 include a tetramerization domain and transactivation domain in the N-terminus and C-terminus ends, respectively (Figure 8) [77]. *TP53* plays a significant part in the regulation of several functions, including DNA damage repair, cell cycle arrest, apoptosis, gluconeogenesis and glycolysis, differentiation, and other cellular processes [78,79]. Mutations are extremely heterogeneous among patients but primarily occur within the gene’s DNA-binding domain, which leads to a loss in the DNA-binding capability [79]. *TP53* mutations typically represent 5–10% of de novo AML cases and 20–35% of therapy-related AML. In mutant p53 proteins, they are unable to effectively bind to DNA, causing atypical target gene transcription [78,80]. Additionally, mutant p53 proteins may form hetero-oligomers with wild-type p53, which can further prevent regulatory functions [78].

*TP53*-mutated AML is classified as a distinct subgroup of AML via the ELN and is associated with a poor prognosis, complex karyotypes, poor response to intensive chemotherapy, and short median OS rates [2,80]. Grob et al. in 2022 [81] studied 2200 patients with either AML or myelodysplastic syndrome with excess blasts (MDS-EB) and detected that 10.5% of them had *TP53* mutations and 76% had bi-allelic *TP53* status. As established in the ELN 2017 adverse-risk category, *TP53*-mutated AML patients see a 2-year OS of 12.8% in *TP53*-mutated AML. Meanwhile, Nong et al. 2024 [78] stated biallelic *TP53* mutations were associated with an extremely poor prognosis, with a 2-year OS of 4% compared to monoallelic mutations with a 2-year OS of 43%. However, both variants are associated with very poor outcomes due to resistance to intensive chemotherapy and HMAs. In general, patients with *TP53*-mutated AML have a median OS of less than six months. The frequent co-mutations of *TP53* with other aberrations further complicate the issue. *TP53* mutations are often associated with specific copy number alterations and the presence of a monosomal karyotype [6]. Subclonal mutations in *TP53*, defined by variant allele frequencies <20%, serve as a significant adverse prognostic marker for patients [82]. In previous studies, patients with subclonal *TP53* in AML were seen to exhibit negative trends in CR, OS, and EFS rates, along with increased resistance to chemotherapy [82,83].

*TP53* mutations carry a poor prognosis because of their resistance to many conventional and standard therapies, including a 10-day regimen of decitabine (DEC10) with venetoclax. In 2021, Kim et al. [80] found that the mutation caused lower response rates and shorter survival. Furthermore, traditional techniques, such as allo-HCT, led to long-term remission in only 10–15% of eligible patients, necessitating the development of novel therapeutic treatments. One prospective novel therapy is APR-246 (eprenetapopt), which works by refolding mutant p53 proteins back into their wild-type conformation. In preclinical trials, it has been shown to induce apoptosis and restrict tumorigenesis in *TP53* mutant leukemic cells [78]. In clinical trials, *TP53*-mutated MDS and AML patients treated with APR-246 and azacitidine saw a CR of 50% and a median OS of 10.8 months, though subsequent studies have not confirmed long-term results, leading to a discontinuation of many trials with this combination [78]. Other therapies are also being explored, including MDM2 inhibitors, which reactivate the p53 pathway by blocking its negative regulator. Combination therapies and immune checkpoint inhibitors, such as pembrolizumab, are also being tested. Despite these efforts, the need for new and effective treatments for *TP53*-mutated AML continues to grow [78].

## 9. *CEBPA*

**Figure 9:**
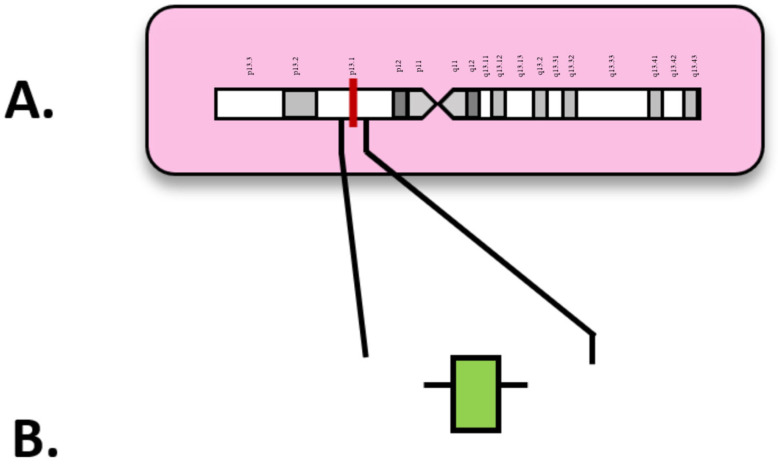
(**A**) Locus of *CEBPA* on 19q13.1; (**B**) *CEBPA* diagram of exon. Modified from McGowan-Jordan, et al., 2020 [35].

**Figure 10:**
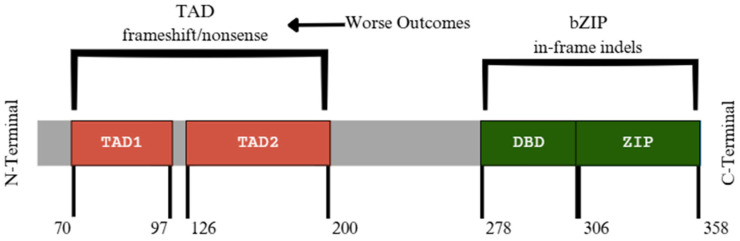
Relative domain locations in C/EBPα protein, with amino acid range for each domain presented. Location of DNA-binding domain (DBD) and the leucine zipper (ZIP) components of bZIP are presented with the transactivation domain (TAD) domain located at the opposite N-terminal end [86]. Modified from Tian and Graf et al., 2014 [86].

*CEBPA* (CCAAT enhancer binding protein alpha) gene mutations are relatively common in patients with AML, with a higher incidence rate observed in Asian countries [84]. The gene itself is located on chromosome 19 (19q13.1) (Figure 9A) and is an important transcription factor involved in granulocyte differentiation, along with roles in metabolism regulation [84]. Also, as seen in Figure 9B, *CEBPA* is made up of just one exon. Incidence rates are 18% in pediatric patients and 10% in adults (Table 1). *CEBPA* mutations frequently take on two main forms, N-terminal frameshift and C-terminal in-frame deletions/insertions, although mutations are not limited to these areas [84]. Both double mutations affecting both the N and C-terminus, as well as single mutations affecting only one terminal, exhibit favorable outcomes. This is particularly true in patients who also display in-frame mutations within the basic leucine zipper (bZIP) domain of *CEBPA*, which is in the C-terminal (Figure 10). This may allow for classifications of AML with *CEBPA* to place greater emphasis on alterations in the bZIP domain rather than single or double mutation categories [84]. Two transactivation domains (TADs) are in the N-terminal; mutations affecting these domains typically show worse outcomes in contrast to bZIP-affected counterparts (Figure 10).

A 2024 multivariable study analyzing both site and allelic status of mutation observed that patients with double-mutation *CEBPA* (dm*CEBPA*) exhibited alterations in both the bZIP and TADs. In rare cases, the double mutation involved two alterations within the bZIP domain [85]. Meanwhile, mutated *GATA2* was found in only 4–12% of patients within subgroups that had non-bZIP insertion/deletion mutations (bZIP indel). In contrast, it was present in 33–39% of patients in other mutation subgroups. Groups defined by the presence of bZIP indel mutations were also observed to have higher rates of Wilms’ tumor gene 1 (*WT1*) co-mutations. This same subgroup was categorized not only by a striking co-mutation profile but also by the younger age of the patient sample.

A study by Wakita et al. (2022) [87] corroborated the conclusion of dm*CEBPA* exhibiting combinations of bZIP mutations and non-bZIP mutations. Though the analysis of this cohort did not include a TAD analysis, the findings of Georgi et al. (2024) [85] may fill in the non-bZIP category. *NPM1* mutations were found in 5.2% of AML patients with *CEBPA*mu in bZIP, and *FLT3*-ITD mutations were present in 4.3% of AML patients with *CEBPA*mu in bZIP. In AML patients with *CEBPA* mutations in the bZIP domain, the co-occurrence of *NPM1* and *FLT3*-ITD mutations was less frequent compared to their rates in the overall AML population.

## 10. Therapeutic Approaches and Treatment Limitations in AML

Given the ELN 2022 and 2024 guidelines, the management of AML has improved significantly with the surfacing of new genetic screening protocols and high-resolution techniques. At the time of diagnosis, considerable care should be taken in conducting comprehensive genetic profiling of the patient to identify defining mutations and characteristics [2]. These include techniques such as conventional cytogenetic techniques and fluorescent in situ hybridization (FISH); NGS in determining mutations in key genes such as *NPM1*, *FLT3*, *TP53*, *RUNX1*, etc.; and OGM to detect structural variants and copy number variants that cannot be found through conventional methods [2,11,23].

The ELN 2022 guidelines categorize patients into favorable, intermediate, or adverse risk groups based on detected cytogenetic abnormalities and mutations [2]. In the ELN 2024 update, multiple revisions can be found regarding older/unfit patients that require less intensive therapies [3]. Given the ELN risk stratification and overall patient fitness, treatment selection is tailored to each patient. In the favorable risk group, where patients are more fit, standard induction therapy is typically chosen. Intensive chemotherapy, for example, 7 + 3, is followed by consolidation and maintenance as needed [2]. For patients receiving less intensive therapies, HMAs such as azacitidine or decitabine combined with venetoclax and standard therapy have shown positive results in response rates and OS [3,46].

Meanwhile, monitoring MRD is vital in determining CR or CR with incomplete hematologic recovery (CRi) for patients of both intensive and non-intensive therapies. For many AML patients, MRD assessment provides a reliable and quantitative method of determining remission status and risk of relapse [2,88]. Multiparametric flow cytometry (MFC) and molecular MRD assessed by qPCR for specific fusion transcripts are two widely used methods of MRD assessment, with MRD detection before allo-HCT being an extremely accurate predictor of relapse [88,89]. In 2025, Ali et al. found that patients who did not experience relapse by day 100 after HCT typically did not display MRD, but those who did were accurately identified at an increased risk of relapse and worse survival. Furthermore, those with pre-HCT MRD cleared MRD by days 70–100 after HCT, but with significantly worse outcomes than MRD-negative patients. Interestingly, more than half of the patients studied ended up relapsing after HCT within 100 days of HCT. However, as expected, these relapses were much more common in pre-HCT MRD patients than those without [89].

Though MRD is a valuable prognostic tool, it is not without its flaws. Relapses are still possible in MRD-negative patients, though rare. As a result, patients may not have complete disease eradication but a level below the threshold of MRD. Inversely, not all MRD-positive patients will relapse. Apart from prognosis, MRD can also serve as a therapeutic tool to determine stem cell transplantation for patients. It has been seen in *NPM1*-mutated AML patients that receiving HCT had no significant benefit for patients who did not display detectable MRD or a >4 log reduction in MRD prior to HCT [90]. As a result, MRD detection can be considered in deciding on HCT for patients with *NPM1*-mutated AML, as well as reserving HCT for patients with an MRD response. MRD remains limited by technical variability, heterogeneous cutoffs, and relapses, even in MRD-negative patients. Overall, these issues complicate standardizations and stress the need for more homogenized assays before MRD can fully guide any therapeutic decision-making.

In less-intensively treated patients, MRD serves as an extremely valuable prognostic tool [47]. Patients treated with azacitidine and venetoclax who then achieved CR or CRi saw that negative MFC-MRD was associated with a significantly better outcome than their counterparts. In an *NPM1*-mutated AML study of patients treated with HMA + venetoclax, MRD negativity saw a better OS, EFS, and lower incidence of relapse than MRD positivity [47,91].

Post-remission strategies are decided by MRD status and initial risk stratification, with patients achieving CR typically consolidated with intermediate-to-high-dose cytarabine [2,91]. Patients with intermediate/adverse risk or positive MRD can benefit from consolidation with allo-HCT. In patients not undergoing transplant or in older patients, maintenance therapy with oral azacitidine (CC-486) is associated with longer OS and relapse-free survival (RFS) [92]. In relapsed/refractory disease management, molecular re-evaluation is important to identify patients suitable for salvage regimens with clinical trial enrollment encouraged, especially those with *TP53* mutations or complex karyotypes [2,93]. Patients with primary refractory AML, defined as a lack of remission after two cycles of induction with at least one cycle of intermediate-dose cytarabine, are unlikely to benefit from further chemotherapy. It is instead encouraged to undergo allo-HCT or clinical trial enrollment [2]. It has been studied that patients who are older, MRD-positive, have a non-favorable risk karyotype, or have incomplete recovery of peripheral counts are all at an increased risk of relapse [2,94].

In patients with relapsed/refractory *FLT3* mutations, gilteritinib showed significantly improved CR rates, OS, and rates of remission than salvage chemotherapy [95]. Enasidenib and ivosidenib are associated with durable remissions and favorable outcomes in *IDH1-* and *IDH2*-mutated relapsed/refractory AML patients. In *IDH1*-mutated patients treated with ivosidenib monotherapy, CR or complete remission with partial hematologic recovery (CRh) is detected at 30.4%, and among those that achieve CR or CRh, 21% saw no residual *IDH1* detection [2,95]. For most patients, after cytoreduction, allo-HCT is encouraged. In older patients, less-intensive treatment, such as HMA with/without venetoclax, is favored [2].

Furthermore, AML exhibits an extensive clonal heterogeneity, which requires different treatment methods as well as the potential for relapses. Multiple studies have shown this clonal structure evolving throughout therapeutic treatment, with NGS studies suggesting that relapsed patients disproportionately have higher mutational risk stratification at diagnosis than those without relapse. This complicates and limits the prognosis and durability of therapeutic response, even in patients in a favorable-risk category [96].

With the expansion of novel combinations of therapies in recent years, multiple adverse events (AEs) have been associated with these agents. Understanding these AEs is thus critical for managing the effects of AML therapies in delivering patient care [97]. In the case of HMA/venetoclax, common grade ≥3 AEs included thrombocytopenia, neutropenia, and infections. Modifications to address these issues include delaying cycles, decreasing venetoclax administration duration per cycle, and dose adjustment of venetoclax. In cases such as prolonged cytopenia, where treatment length is indefinite, the length of cycles may be extended to 5–6 weeks, and the duration and dosage of HMAs decreased [97,98]. Also reported, but rare, is tumor lysis syndrome (TLS) with HMA/venetoclax in 1% of patients. Modifications can include a reduction in WBC <25 × 10^9^/L with hydroxyurea before treatment and administering venetoclax with a ramp-up to target dose approach.

In *IDH* inhibitors enasidenib and ivosidenib, AEs can include differentiation syndrome (DS), leukocytosis, and QTc prolongation [99]. DS is characterized by dyspnea, fever/hypotension, weight gain, acute renal failure, or pleuropericardial effusions and is an extremely important, and potentially fatal, AE. The incidence of DS with *IDH* inhibitors ranged from 13 to 25%, though fatalities were rarely recorded [97]. In *FLT3* inhibitor midostaurin, GI toxicities such as nausea, vomiting, and diarrhea, as well as cardiac toxicities (primarily in older patients), have been recorded [100,101]. Gilteritinib has also been reported to have AEs of DS, QTc prolongation, and posterior reversible encephalopathy syndrome (PRES). DS was seen in 3% of patients, with PRES in 1% of patients necessitating the immediate discontinuation of gilteritinib once confirmed [97,102].

## 11. Future Directions of AML Research

**Figure 11:**
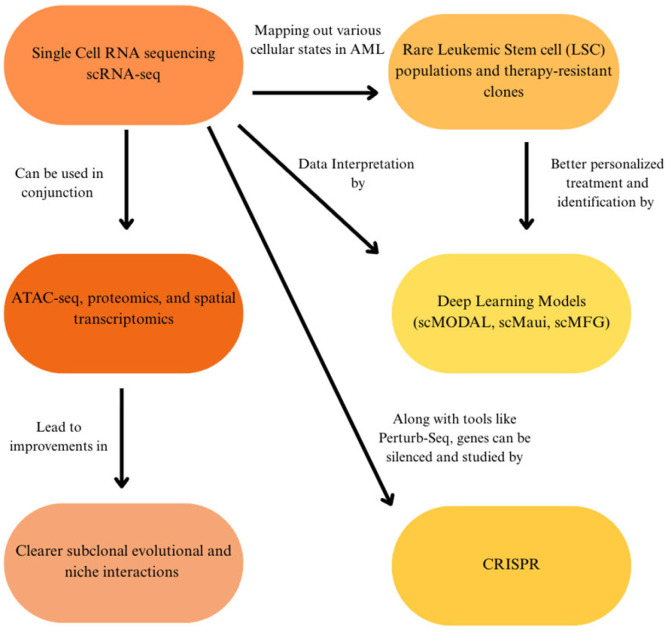
Technological advancements in AML research, with predictions on how techniques can be consolidated. Incorporation of multiple technologies in improving patient health, identification, and personal treatment.

In the next few years, AML research is expected to greatly benefit from advances in high-resolution genomics, functional screening, and computational analysis. One of the most promising developments is in the growth of single-cell sequencing in the heterogeneity of AML (Figure 11). One example, single-cell RNA sequencing (scRNA-seq), is an established technique that has already been able to map out various cellular states in AML, such as rare leukemic stem cell (LSC) populations and therapy-resistant clones [103]. The next step will likely involve combining scRNA-seq with further methods, such as ATAC-seq, proteomics, and spatial transcriptomics [104]. As a result, the integration could offer a much clearer image of how subclones evolve over time and how they interact in their native microenvironment.

Furthermore, it will most likely be seen that computational tools will play an increasingly important role in interpreting the data generated by these experiments. New deep learning models have been developed specifically for single-cell and multi-omics data, enabling researchers to find meaningful biological patterns while minimizing batch effects [105]. scMODAL, a deep learning model, combines multi-modal single-cell datasets like scRNA-seq and proteomics through neural networks and generative adversarial networks to eliminate variation while preserving biological signals across modalities [105]. Another model, scMaui, has been found to effectively balance transcriptomic and proteomic modalities while simultaneously correcting for batch effects and enhancing downstream analyses like cell-type classifications [106]. Meanwhile, the scMFG model employs feature grouping and interpretable matrix-factorization in integrating multi-omics datasets, retaining biological interpretability and revealing rare cell types even in technically noisy datasets [107]. Using single-cell multi-omics data, these models now allow researchers to better identify LSC populations, monitor clonal evolution, and ultimately facilitate a more personalized and data-driven treatment strategy for individual patients.

Integrated single-cell multi-omics that merge scRNA-seq, ATAC-seq, mitochondrial genotyping, and clonal tracking continue to improve our understanding of AML subclones and LSCs. A 2024 review highlighted how platforms like CITE-seq and TARGET-seq enable high-resolution profiling of transcriptional, surface protein, and mutational states within individual cells, and offer a critical insight into therapy resistance mechanisms [108]. In a different 2024 study focusing on spatial multi-omic profiling, researchers applied spatial transcriptomics in studying post-transplant AML bone marrow, revealing distinct “immune hubs” in tumor microenvironments, opening a valuable pathway into the mechanisms of relapse and remission maintenance after donor lymphocyte infusion [109]. Work with co-detection by indexing (CODEX), especially with scRNA-seq, has further mapped out how mesenchymal stromal cells (MSCs) and leukemic blasts co-localize within spatially organized microenvironments, creating niches that may fuel resistance and persistence (Figure 11) [110]. Single-cell and spatial multi-omics thus offer a straightforward way to resolve the challenge of clonal heterogeneity by enabling the high-resolution tracking of resistant subclones through multiple modalities of detection and treatment (Figure 11).

However, single-cell and spatial multi-omics remain a costly tool, technically restraining, and limited to a few research centers, confining its clinical impact [103,104,105,106,107,108,109,110]. Their successful translation will rely on improvements in both affordability and scalability. Concurrently, the growth of genome-wide functional screens using CRISPR has become much more accessible. Tools like Perturb-seq, which combine CRISPR with scRNA-seq, allow for the visualization of primary AML cells, allowing researchers to systematically silence and activate genes to study their impact on cell survival and drug sensitivity [111]. Meanwhile, non-invasive monitoring procedures are improving every year, like the development of circulating tumor DNA (ctDNA)-based AML assays to detect minimal residual disease (MRD) with high sensitivity. Tumor-informed methods, such as AML-CAPP-Seq, can illuminate mutations in tracking ctDNA progression and have demonstrated detection thresholds < 0.01%, and even tumor-agnostic approaches, like methylation-based assays and fixed mutation panels, show potential in routine MRD monitoring [112]. Collectively, these approaches can eventually reduce the need for repeated bone marrow biopsies as well as earlier detection of relapse. Looking forward, these advances are expected to thoroughly refine and deepen our understanding of the landscape of AML and the ability to identify new therapeutic targets.

## 12. Clinical Implications and Changing Approaches to Patient Care

**Figure 12:**
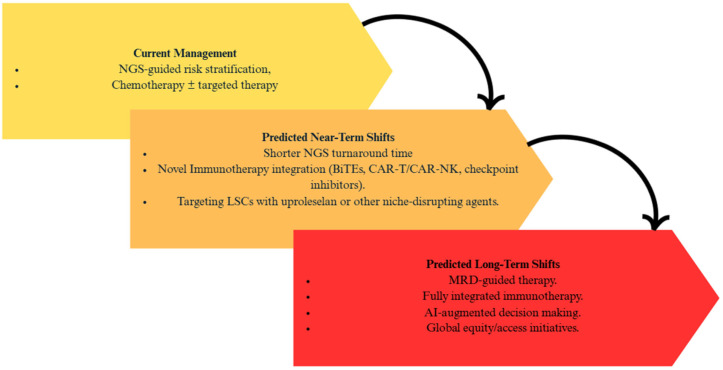
Evolving paradigm of AML management. From current methods of management to insights into long-term shifts in treatment and disease management.

As genomic technologies develop and become faster, more affordable, and further incorporated into clinical practice, the management of AML is likely to undergo substantial changes in the next three to five years. One of the most immediate shifts will be in the timing and use of genomic data. Turnaround times for NGS now approach 48–72 h in some institutions; clinicians may soon have full mutational profiles in hand before even starting induction therapy (Figure 12) [113]. This may support earlier, more precise treatment decisions, for example, starting a FLT3 inhibitor up front in a newly diagnosed FLT3-ITD-positive patient, or avoiding standard 7 + 3 chemotherapy altogether in favor of a venetoclax-based regimen in older adults or those with poor-risk mutations [2]. These shifts are consistent with previously discussed evidence where the ability to profile mutational abnormalities early is shown to influence both prognosis and initial treatment selection, justifying prioritization of faster, clinically actionable NGS (Figure 12).

Immunotherapy, more broadly, is also gaining ground in AML, although progress has been slower than in other forms of cancer. Bispecific T-cell engagers (BiTEs), antibody–drug conjugates, chimeric antigen receptor (CAR)-T and CAR-NK cells, and checkpoint inhibitors are all being investigated for use in relapsed/refractory AML (Figure 12). Recent efforts have focused on improving the specificity and safety of these therapies, for example, by designing dual antigen CARs or by incorporating logic-gated controls to prevent off-target toxicity [114]. These novel immunotherapy strategies place a particular emphasis on the integration of modern immune-based strategies to improve outcomes in adverse-risk and MRD-positive patients. This corresponds with our discussion that MRD detection before all-HSCT is an extremely accurate predictor of relapse and poor survival rates [88,89]. Therefore, the prioritizing of immunotherapy approaches aimed at clearing residual disease directly addresses these needs. Over the next few years, these agents may find themselves in a more front-stage role in combination regimens, especially for clearing MRD after initial therapy or treating patients with high-risk molecular profiles.

Fresh endeavors to target the leukemia stem cell (LSC) population, a key driver of relapse, are also gaining momentum. Agents such as uproleselan, an E-selectin inhibitor, focus on disrupting the protective bone marrow that shelters LSCs from chemotherapy. A phase 3 trial evaluating uproleselan jointly with cytarabine-based chemotherapy is currently underway and has the possibility to shift the model for addressing minimal residual disease and chemoresistance [115]. Targeting LSC-specific pathways may better improve the elimination of residual disease post-remission and reduce the risk of relapse, especially when integrated into immunotherapy regimens or MRD-guided treatment strategies (Figure 12).

Looking ahead, equity in accessing these innovations proves a significant concern. The most advanced diagnostic and therapeutic technologies are still limited to academic centers in high-income countries. However, several groups are working on simplified, lower-cost MRD assays and sequencing protocols that could be enacted in community hospitals or lower-resource settings. Further advancements in automation, cloud-based AI interpretation, and decentralized testing could aid in making precision medicine a reality for more patients worldwide [116].

**Figure 13:**
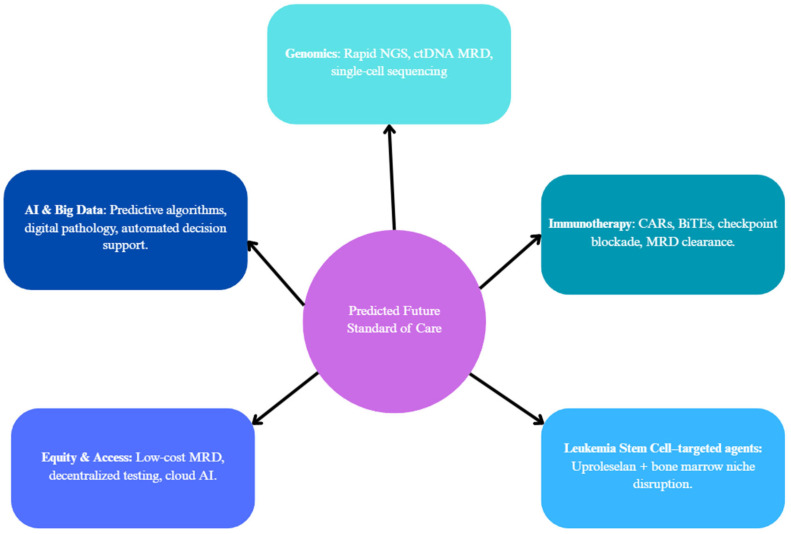
Future standard of care in AML: integration of genomics, immunotherapy, AI, and equity. Predicted directions of change in major future components of care.

Implementation of big data and artificial intelligence into AML management promises to further improve efforts in diagnostics and personalized therapy. Machine learning algorithms trained on large genomic and clinical datasets have already demonstrated their potential to predict treatment response and survival outcomes, even outperforming traditional risk scores in some models (Figure 13) [117]. Furthermore, digital pathology tools utilizing deep learning can aid in a variety of aspects, such as helping to quantify blast burden, assess bone marrow morphology, or even detect early signs of relapse [118]. As these systems develop further and become more accessible, they may allow for a wider outreach of precision oncology by reducing the burden on overextended human expertise, particularly in community or resource-limited settings. These proposed shifts are justified by the complexity and heterogeneity of AML treatment responses previously outlined in this review, where mutational risk stratification and MRD monitoring alone were insufficient in a clear outcome prediction for many patients [2,88,90]. Thus, modern technologies, such as AI, show potential in improving predictive accuracies, especially in lower-income and resource-limited settings [Figure 13].

To prepare for these shifts in practice, the field should increase its focus on prioritizing a set of critical research goals. These can include the standardization and clinical validity of ctDNA MRD assays, integration of single-cell technologies into decision-making workflows, and the expansion of immunotherapy trials into maintenance and frontline settings (Figure 13). At the same time, current AI and digital pathology models are trained primarily in high-income regions, requiring broader validation to minimize algorithmic bias and maximize applicability to more diverse and clinical settings. Addressing infrastructure and training gaps in underdeveloped regions is also of high importance in ensuring that these tools reach all patients, not just those treated at top-tier institutions.

## 13. Discussions and Conclusions

This review functionally integrates AML genomics with detection modalities (NGS, FISH, and OGM) and different care pathways. Going over all mutation groups, evidence for clinical utility in the changing of risk groups is strong for *NPM1* (including MRD), *FLT3* (ITD allelic ratio), and *IDH1*/2 (targeted inhibitors); moderate for *ASXL1*, *RUNX1*, and adverse structural lesions (*KMT2A*, *NUP98*, inv(3)/*MECOM*); and limited/heterogeneous for several rarer alterations. Our unique contribution is a modality to management map, linking assay design (panel coverage, hotspot bias, ITD allelic-ratio detection, CN/LOH assessment for *TP53*) to concrete risks of misclassification and missed therapeutic eligibility, which is summarized in Table 2, applied across the gene-specific sections, and operationalized in the clinical workflow sections. These conclusions align with recent ELN/ICC recommendations and contemporary reviews, while also extending the prior work by recognizing where detection gaps most often alter the ELN/ICC category, MRD interpretation, and therapy selection (see Figure 11, Figure 12 and Figure 13).

In this review, while an overview of recent advances in risk stratification and therapeutic approaches was addressed, several limitations of the studies should be acknowledged. Many referenced studies included relatively small patient populations and often within single-center settings, limiting statistical power and the generalizability of results. Furthermore, much available evidence is derived from retrospective analyses of heterogeneous populations and variable treatment administrations, causing potential partiality compared to prospective randomized trials.

**Figure 14:**
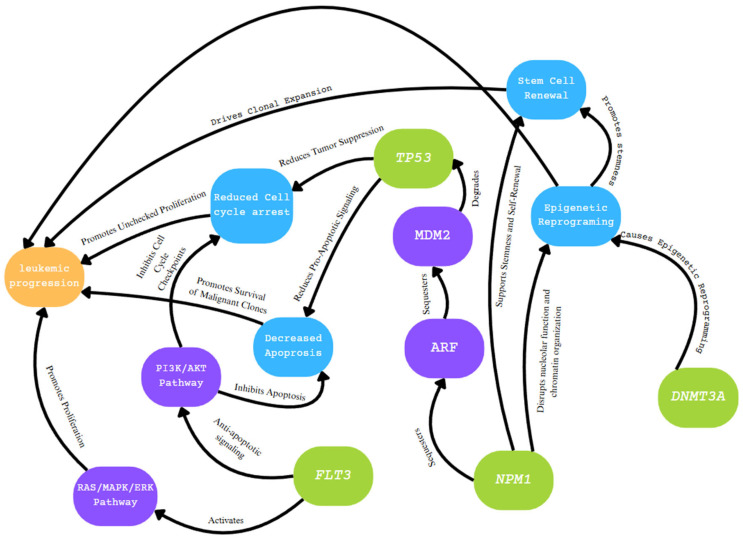
Overview of overlapping and related effects caused by dysfunction in *NPM1*, *FLT3*, *TP53*, and *DNMT3A* and related pathways. Irregular gene types are labeled in green; associated pathways and proteins are labeled in purple; induced effects leading to leukemic progression are labeled in blue [33,53,61].

AML remains a complex and heterogeneous hematologic malignancy that frequently spurs amended and improved clinical updates. Many mutations that are present within varying cases of AML include overlapping pathways that induce negative effects in regard to leukemic progression (Figure 11). Previously mentioned pathways, such as the RAS pathway, PI3K/AKT pathways, and others, have downstream consequences that are shared between irregularities in certain genes, such as *NPM1*, *FLT3*, *DNMT3A*, and *TP53* (Figure 14). The introduction of techniques such as NGS and OGM in recent decades has allowed for clinical diagnoses of AML to be conducted more efficiently and accurately, along with significant progress in treatment and overall prognosis. Understanding the importance of genetic mutations in AML can definitively shape the prognosis of a patient and inform critical therapeutic decisions.

## Figures and Tables

**Table 2:** Comparative performance and clinical implications of NGS, FISH, and optical genome mapping in AML.

Attribute	NGS (Short-Read Targeted Panels/WES/WGS)	FISH	Optical Genome Mapping (OGM)
Primary targets	SNVs, small indels; some CNVs/SVs depending on design	Known rearrangements, aneuploidies, and copy number at targeted loci	Large/complex SVs (translocations, inversions, insertions, deletions, CNVs); repeat expansions; genome-wide
Sensitivity	High for SNVs/indels; VAF often ≥1–5% for panels; SV/CNV sensitivity depends on assay/bioinformatics [22,23,24,25,26,27,28,29,30]	High for targeted loci if probe exists; detects mosaicism to ~5–10% depending on lab [28]	Genome-wide SV detection; claims down to ~5% VAF for mosaic/heterogeneous samples (assay-dependent) [11,12]
Specificity	High with orthogonal confirmation; false positives from artifacts/low-complexity regions; panel design matters [22,23,24,25,26,27,28,29,30]	High for predefined targets; interpretation can be confounded by signal overlap/signal noise [28]	High for large SVs; specificity impacted by complex duplications/repeats; orthogonal confirmation recommended [11,12]
Resolution	Single-base for SNVs/indels; exon-level to gene-level for CNVs/SVs depending on depth/algorithm [22,23,24,25,26,27,28,29,30]	Probe-level (typically 100–200 kb around target; break-apart or fusion probes) [28]	Kilobase-scale mapping of long molecules; resolves structure/orientation and partners for many SVs [11,12]
Prior knowledge needed	Not for broad panels/WES/WGS; targeted panels predefine genes/hotspots	Yes—probe must be designed for suspected locus/rearrangement	No (genome-wide), unbiased for SV discovery
Advantages	Detects recurrent AML SNVs/indels (e.g., *NPM1*, *DNMT3A*, *FLT3*); scalable; actionable variant interpretation frameworks exist [22,23,24,25,26,27,28,29,30]	Rapid, cost-effective for confirming known rearrangements (e.g., *MECOM*, *RUNX1*); single-cell resolution	Single assay for all major SV classes; clarifies complex/cryptic rearrangements and fusions; complements NGS [11,12]
Limitations	Short reads miss/under-resolve complex SVs, repeats; panel bias and variable exon coverage; SV calling requires optimized pipelines [22,23,24,25,26,27,28,29,30]	Limited scope (only where probes exist); cannot genome-wide screen; lower structural detail	Limited for small variants (SNVs/indels); VAF cutoffs need clinical context; orthogonal confirmation often needed [11,12,31]
Typical VAF/abundance limits	Panels often call ≥1–5% VAF for SNVs (lab-dependent); SV/CNV thresholds vary [22,23,24,25,26,27,28,29,30]	Mosaic detection depends on probe/assay (~5–10% typical) [28]	Reported ~5% for SVs in cancer samples; interpret with disease-specific rules (e.g., TP53 in ICC) [12,31]
Best use cases in AML	Mutational profiling for diagnosis/risk (e.g., *NPM1*, *FLT3*-ITD/TKD, *DNMT3A*, *ASXL1*, *TP53*); MRD with specialized assays	Rapid confirmation of specific rearrangements (e.g., *MECOM* break-apart), aneuploidies, and hallmark fusions	Discovery/refinement of karyotype-scale abnormalities, cryptic/complex rearrangements, fusion partners; complement to NGS
Turnaround (typical)	Days (panels) to 1–2 weeks (WES/WGS); lab-specific	24–72 h for targeted probes	Days to ~1 week; lab-specific
Notes on validation	Confirm key calls (esp. low VAF or SVs) with orthogonal method (ddPCR, Sanger, FISH)	Use with orthogonal methods for breakpoint/partner resolution	Confirm clinically actionable SVs with NGS/FISH or PCR, per lab policy

## Data Availability

Not applicable.
